# Cross-sectional study of IgG antibody levels to invasive nontyphoidal *Salmonella* LPS O-antigen with age in Uganda

**DOI:** 10.12688/gatesopenres.13034.1

**Published:** 2019-06-26

**Authors:** Lisa Stockdale, Angela Nalwoga, Stephen Nash, Sean Elias, Gershim Asiki, Sylvia Kusemererwa, James J. Gilchrist, Robert Newton, Calman A. MacLennan

**Affiliations:** 1 Department of Paediatrics, University of Oxford, Oxford, OX3 7LE, UK; 2 MRC/UVRI and LSHTM Uganda Research Institute, Entebbe, Uganda; 3 Department of Infectious Disease Epidemiology, London School of Hygiene and Tropical Medicine, London, WC1E 7HT, UK; 4 Nuffield Department of Medicine, University of Oxford, Oxford, OX3 7DQ, UK; 5 Clinical Epidemiology, University of York, York, UK

**Keywords:** non-typhoidal salmonella, NTS, antibody, Uganda

## Abstract

Invasive nontyphoidal *Salmonella* (iNTS) disease is a major cause of deaths among children and HIV-infected individuals in sub-Saharan Africa. Acquisition of IgG to iNTS lipopolysaccharide (LPS) O-antigen in Malawi in early childhood corresponds with a fall in cases of iNTS disease suggesting that vaccines able to induce such antibodies could confer protection. To better understand the acquisition of IgG to iNTS in other African settings, we performed a cross-sectional seroepidemiological study using sera from 1090 Ugandan individuals aged from infancy to old age. Sera were analysed for IgG to LPS O-antigen of *S*. Typhimurium and *S*. Enteritidis using an in-house ELISA. Below 18 months of age, most children lacked IgG to both serovars. Thereafter, specific IgG levels increased with age, peaking in adulthood, and did not wane noticeably in old age. There was no clear difference in antibody levels between the sexes and the few HIV-infected individuals in the study did not have obviously different levels from uninfected subjects. While IgG to iNTS is acquired at a younger age in Malawian compared with Ugandan children, it is not clear whether this is due to differences in the populations themselves, their exposure to iNTS, or variations between assays used. In conclusion, there is a need to develop a harmonised method and standards for measuring antibodies to iNTS across studies and to investigate acquisition of such antibodies with age across different sites in sub-Saharan Africa.

## Introduction

Invasive non-typhoidal *Salmonella* (iNTS) disease is principally caused by serovars *S*. Typhimurium and *S*. Enteritidis and is thought to be responsible for up to 680,000 deaths annually, with Africa accounting for more than half of cases ^1^. Much of this burden is in children under 5 years and HIV-infected adults. In view of this major global burden of disease, and rapid emergence of multidrug resistant iNTS strains ^2^, development of a vaccine is increasingly vital ^3^.

Studies in Malawian children indicate that anti- *S. Typhimurium* antibodies, notably IgG to O-antigen of LPS and flagellin, and serum bactericidal activity rises rapidly with age in the first few years of life corresponding with a fall in cases of iNTS disease ^4, 5^. One study found a positive correlation between serum bactericidal assay (SBA) killing and acquisition of anti-LPS IgG ^5^. However, there is no standardised assay for measurement of iNTS-specific IgG, and the clinical significance of the iNTS SBA is unknown. Given that incidence of iNTS disease drops in children over 2 years, it has been suggested that a rise in specific antibodies and bactericidal activity correlates with protection. This hypothesis is complicated by the observation that among HIV-infected Malawian adults, high LPS-specific IgG was associated with a lack of *in vitro* bacterial killing ^6^.

## Methods

In a cross-sectional study, we investigated NTS-specific antibody responses in the rural Ugandan General Population Cohort (GPC) ^7^. Levels of IgG against serovars *S*. Typhimurium and *S*. Enteritidis LPS O-antigens were measured using a standardised in-house ELISA in stored sera from a cross-section of 1,090 Ugandans of all ages, 10 of whom were HIV-infected. Sera from adults (≥ 16 years) were collected from January 2014 to November 2015, and children (<16 years) from January 2016 to November 2017. Antibody units (AU) were calculated using Gen5 software (version 2.0) using a five-parameter logistic (5PL) curve generated with a standard serum from an iNTS-exposed individual. Sera were defined as seronegative if below the lower limit of detection (4 AU for *S.* Typhimurium and 5 AU for *S.* Enteritidis) at 1:100 serum dilution.

Written informed consent for the use of clinical records and biological samples for research purposes was obtained from all GPC participants following Uganda National Council of Science and Technology guidelines. Ethical approval for the use of samples for this study was obtained from The UVRI Research and Ethics Committee and from the Uganda Council for Science and Technology (Ref: GC/127/19/10/710).

## Results and discussion

**Figure 1.**
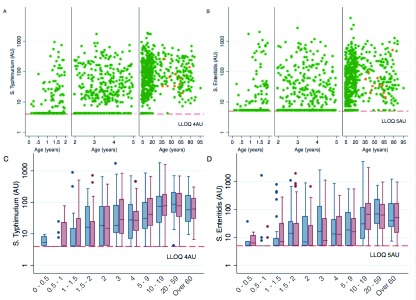
Plots showing antibody units (AU) for S. Typhimurium ( **A** and **C**) and S. Enteritidis ( **B** and **D**) by age. Orange dots indicate HIV infected individuals. ( **A**, **B**). Females are indicated in red and males in blue ( **C**, **D**). The box shows the interquartile range (IQR) with middle line representing the median. The whiskers represent the adjacent values, defined as 1.5 × IQR from the edge of the box, with values outside this range shown individually. LLOQ, lower limit of quantification.

In this assay, overall O-antigen seropositivity was 82% for *S.* Typhimurium, and was 70% for *S.* Enteritidis. Levels of antibody were undetectable in at least 50% of children until 18 months for both serovars and a similar pattern of increasing IgG level was observed with increasing age ( Figure 1A, B). There were no observable differences in antibody levels by sex ( Figure 1C, D). HIV-infected individuals did not have notably high IgG antibody responses, although the study was not powered to demonstrate this.

Although performed using a flow cytometric assay, previously published data from Malawi suggest that NTS-specific IgG is present in the majority of children throughout infancy ^4^, contrasting with our results from Uganda. This could be due to variation in exposure to iNTS in Uganda compared to Malawi, or differences in assays. However, burden of, and exposure to, iNTS disease in Uganda is not well understood. A standardised assay is key to understanding variation in exposure across geographic locations to support vaccine development.

## Data availability

Open Science Framework: Invasive Non-Typhoidal Salmonella serology in Uganda. https://doi.org/10.17605/OSF.IO/68BYT ^8^.

This project contains the age, sex, antibody levels, HIV status and *Salmonella* status of each participant.

Data are available under the terms of the Creative Commons Zero "No rights reserved" data waiver (CC0 1.0 Public domain dedication).

## References

[1] Ao TT Feasey NA Gordon MA: Global burden of invasive nontyphoidal *Salmonella* disease, 2010. *Emerg Infect Dis.* 2015;21(6):941–949. 10.3201/eid2106.140999 25860298 PMC4451910

[2] Oneko M Kariuki S Muturi-Kioi V: Emergence of Community-Acquired, Multidrug-Resistant Invasive Nontyphoidal *Salmonella* Disease in Rural Western Kenya, 2009-2013. *Clin Infect Dis.* 2015;61 Suppl 4:S310–6. 10.1093/cid/civ674 26449946

[3] Gilchrist JJ MacLennan CA: Invasive Nontyphoidal *Salmonella* Disease in Africa. *EcoSal Plus.* 2019;8(2). 10.1128/ecosalplus.ESP-0007-2018 30657108 PMC11573285

[4] MacLennan CA Gondwe EN Msefula CL: The neglected role of antibody in protection against bacteremia caused by nontyphoidal strains of *Salmonella* in African children. *J Clin Invest.* 2008;118(4):1553–1562. 10.1172/JCI33998 18357343 PMC2268878

[5] Nyirenda TS Gilchrist JJ Feasey NA: Sequential acquisition of T cells and antibodies to nontyphoidal *Salmonella* in Malawian children. *J Infect Dis.* 2014;210(1):56–64. 10.1093/infdis/jiu045 24443544 PMC4054899

[6] Maclennan CA Gilchrist J Gordon MA: Dysregulated humoral immunity to nontyphoidal *Salmonella* in HIV-infected African adults. *Science.* 2010;328(5977):508–512. 10.1126/science.1180346 20413503 PMC3772309

[7] Asiki G Murphy G Nakiyingi-Miiro J: The general population cohort in rural south-western Uganda: a platform for communicable and non-communicable disease studies. *Int J Epidemiol.* 2013;42(1):129–141. 10.1093/ije/dys234 23364209 PMC3600628

[8] Stockdale L: Invasive Non-Typhoidal *Salmonella* serology in Uganda.2019 10.17605/OSF.IO/68BYT

