# Supplementary material for: Cross-sectional study of IgG antibody levels to invasive nontyphoidal Salmonella LPS O-antigen with age in Uganda
Source: Gates Open Res. 2019 Jun 26;3:1501. [Version 1] doi: 10.12688/gatesopenres.13034.1 (PMC6640003; doi:10.12688/gatesopenres.13034.1)
Supplement: Peer review history

# Peer review: Reviewer response for version 1

**DOI:** 10.21956/gatesopenres.14147.r27466

**Published:** 2019-07-30

**Authors:** Michelo Simuyandi

**Affiliations:** 1 Enteric Disease and Vaccine Research Unit, Centre for Infectious Disease Research in Zambia (CIDRZ), Lusaka, Zambia

The article describes an important aspect of research which needs more data. Knowing when children are exposed to salmonella infection can inform schedules for vaccination and other control interventions.

1. The aim of the study should be clear, given the limitation in the standardized method for immunological assessment (I suspect it was to determine the kinetics of antibody acquisition by age). The differences in assays could be discussed in the limitations and maybe how the difference can affect the date explained as well. 
2. The assay is not described (just called in house) - reviewers could have advised how best to analyse or interpret the data. Did they try to do an EQA with another lab that used a different method to maybe determine concordance?
3. While the infants in this study have antibodies much later than Malawi, it is not clear if the mean concentrations increase with time or if it is just the number(%) (i.e. positivity rates) of participants with antibodies that increase over time. The authors could have compared the reported concentrations between the countries.

I confirm that I have read this submission and believe that I have an appropriate level of expertise to confirm that it is of an acceptable scientific standard, however I have significant reservations, as outlined above.

# Peer review: Reviewer response for version 1

**DOI:** 10.21956/gatesopenres.14147.r27468

**Published:** 2019-07-24

**Authors:** Scott M. Baliban

**Affiliations:** 1 Center for Vaccine Development and Global Health (CVD), University of Maryland School of Medicine, Baltimore, MD, USA

This study aims to assess the presence of serum IgG to S. Enteritidis and Typhimurium by age and sex in a broad Ugandan cohort. It is of value because the sero-epidemiology of NTS in Uganda, as opposed to other African countries, is not well understood.

1. More detail is needed for the in-house ELISA. Presumably, it was conducted with purified OPS, as with other studies from this group (e.g. Fiorino *et al.*, 2017 ^1^). At the moment, it is not clear whether the serum IgG responses are directed against the OPS (and therefore serogroup B/D-specific) as opposed to the core oligosaccharide or lipid A (all other NTS). What is an Antibody Unit?
2. The authors mention that the lack of seroconversion to OAg-specific IgG in Ugandan children could be due to variation in exposure to NTS. To further support this hypothesis, it would be interesting to assess the kinetics of IgM since both anti-Typhimurium IgG and IgM can demonstrate bactericidal activity (Goh *et al.*, 2016 ^2^). If these titers are available, it would add a thought-provoking element to the data set.
3. Please state the statistical test used to compare groups.
4. Serovars do not need to be italicized.
5. Panels A and B are blurry, and the font size for the axes is small. Please add a label to the x-axis of panels C and D.

I confirm that I have read this submission and believe that I have an appropriate level of expertise to confirm that it is of an acceptable scientific standard, however I have significant reservations, as outlined above.

## References

[1] : Immunogenicity of a Bivalent Adjuvanted Glycoconjugate Vaccine against Salmonella Typhimurium and Salmonella Enteritidis. *Front Immunol*.2017;8: 10.3389/fimmu.2017.00168 168 10.3389/fimmu.2017.00168 28289411PMC5326758

[2] : Bactericidal Immunity to Salmonella in Africans and Mechanisms Causing Its Failure in HIV Infection. *PLoS Negl Trop Dis*.2016;10(4) : 10.1371/journal.pntd.0004604 e0004604 10.1371/journal.pntd.0004604 27057743PMC4825999

# Peer review: Reviewer response for version 1

**DOI:** 10.21956/gatesopenres.14147.r27467

**Published:** 2019-07-17

**Authors:** Ruklanthi de Alwis

**Affiliations:** 1 Viral Research and Experimental Medicine Centre, SingHealth-Duke NUS, Singapore, Singapore; 2 Programme in Emerging Infectious Diseases, Duke-NUS Medical School, Singapore, Singapore

The study investigates the sero-prevalence of iNTS, serovar Typhimurium and Enteritidis in Uganda. The study further describes the sero-epidemiology by age and sex of these pathogens in Uganda. This is a very useful study since very little is known about the basic epidemiology of iNTS in the Uganda population.

- Since the authors’ main explanation for the observed differences in sero-prevalence between Malawi and Uganda are due to differences in the experimental assays used to quantify O-antigen IgG, it would be good to give more details of this in-house ELISA assay. 
- Since some of the authors from the Malawi study are also authors of the present study, is it not possible to run some of the Uganda sera (probably the sera in the less than 18 months age group) on the older flow-cytometry technique described in the MacLennan *et al.* (2008 ^1^) paper?
- I agree with the authors that very little is known about the most commonly circulating and disease-causing serovars of NTS in Uganda. However, it might be good to mention one or two current literatures, such as Afema *et al.* (2016 ^2^), where they show that neither *S. *Typhimurium nor *S. *Enteritidis are commonly isolated NTS serovars in Uganda (while in Malawi, Typhimurium and Enteritidis together make up over 90% of NTS cases (Feasey *et al.*, 2015 ^3^)).
- It wouldn’t hurt to show a graph of the O-antigen IgG data in HIV-infected and age-matched healthy individuals.

 

 Minor comments:

- Please add x-axis titles to Figure 1C and 1D. 
- Font labelling on panels 1A and 1B is small. Increase the font size on Figure panels 1A and 1B to match the font size of panels 1C and 1D.
- State the statistical test used to compare O-antigen IgG responses in females and males in Figure 1C and 1D.

I confirm that I have read this submission and believe that I have an appropriate level of expertise to confirm that it is of an acceptable scientific standard.

## References

[1] : The neglected role of antibody in protection against bacteremia caused by nontyphoidal strains of Salmonella in African children. *J Clin Invest*.2008;118(4) : 10.1172/JCI33998 1553-62 10.1172/JCI33998 18357343PMC2268878

[2] : Potential Sources and Transmission of Salmonella and Antimicrobial Resistance in Kampala, Uganda. *PLOS ONE*.2016;11(3) : 10.1371/journal.pone.0152130 10.1371/journal.pone.0152130 PMC480120526999788

[3] : Three Epidemics of Invasive Multidrug-ResistantSalmonella Bloodstream Infection in Blantyre, Malawi, 1998–2014. *Clinical Infectious Diseases*.2015;61(suppl 4) : 10.1093/cid/civ691 S363-S371 10.1093/cid/civ691 PMC459693026449953

# Peer review: Reviewer response for version 1

**DOI:** 10.21956/gatesopenres.14147.r27470

**Published:** 2019-07-09

**Authors:** Qingke Kong

**Affiliations:** 1 University of Florida, Gainesville, FL, USA

This is an ELISA assay to measure the antibodies against O-antigen from S. Typhimurium and Enteritidis from clinical sera collected from patents in Uganda. I do not have scientific questions about this assay, but hope in the future, that the authors could establish a standardized method to figure out the reason for the controversy obtained from two locations.  

 Some minor grammar errors:

1. Please add a comma between “disease” and “suggesting” in: “…corresponds with a fall in cases of iNTS disease suggesting that vaccines able to…”.
2. A comma or conjunction words were missing in: “There was no clear difference in antibody levels between the sexes and the few HIV-infected individuals in the study did not have obviously different levels from uninfected subjects”.
3. “Typhimurium” does not need to be italic.

I confirm that I have read this submission and believe that I have an appropriate level of expertise to confirm that it is of an acceptable scientific standard.

# Response

**Published:** 2019-07-09

**Authors:** Sean Elias

Thank you for your comments. I can confirm that we will be publishing the optimised standardised ELISA protocol in the near future and will fully address the issues that you have highlighted.
